# Retrospective analysis of vector-borne infections in dogs after travelling to endemic areas (2007–2018)

**DOI:** 10.1016/j.vpoa.2019.100015

**Published:** 2019-06-22

**Authors:** Ingo Schäfer, Maria Volkmann, Pamela Beelitz, Roswitha Merle, Elisabeth Müller, Barbara Kohn

**Affiliations:** a Clinic for Small Animals, Faculty of Veterinary Medicine, Freie Universität Berlin, Oertzenweg 19 b, 14163, Berlin, Germany; b Institute of Veterinary Epidemiology and Biostatistics, Freie Universität Berlin, Oertzenweg 19 b, 14163, Berlin, Germany; c Chair for Experimental Parasitology, Faculty of Veterinary Medicine, Ludwig-Maximilians-Universität Munich, Leopoldstraße 5, 80802, Munich, Germany; d Laboklin GmbH & Co.KG, Steubenstraße 4, 97688, Bad Kissingen, Germany

**Keywords:** Ab, antibody, Ag, antigen, DNA, deoxyribonucleic acid, ELISA, enzyme linked immunosorbent assay, IFAT, indirect immunofluorescence test, PCR, polymerase chain reaction, Arthropod-transmitted infections, Vector-borne diseases, Laboratory diagnostics, Germany

## Abstract

Canine vector-borne infections gained in importance in Germany due to growing tourist traffic, the increased import of dogs from abroad and the changing of climatic conditions. The Mediterranean region and southeastern Europe are geographical areas where pathogens such as *Leishmania* (*L*.) *infantum*, *Hepatozoon* (*H.*) *canis*, *Ehrlichia* (*E.*) *canis*, *Anaplasma* (*A.*) *platys* and *Dirofilaria* (*D.*) spp. are endemic. Meanwhile, *Babesia* (*B.*) spp. and *A. phagocytophilum* are present in central and western Europe. The objective of this retrospective study was to evaluate whether dogs were exposed to a corresponding risk of infection when travelling to regions in the Mediterranean area and southeastern Europe, which are endemic for these pathogens.

Medical records and laboratory test results of 303 dogs that travelled to 14 countries endemic for the mentioned canine vector-borne pathogens and that were presented to the Small Animal Clinic at Freie Universität Berlin between 2007 and 2018 were retrospectively reviewed. A total of 1174 test results from external laboratories were descriptively analysed including 525 test results of direct and 649 of indirect determination methods.

Overall, 13% of the tested dogs (40/303) were positive for at least one pathogen. Concurrent infections with two pathogens were detected in 1% of the dogs (4/303). The positive results were: *E. canis* 8% (18/231 dogs; Polymerase chain reaction [PCR] 3/73, indirect immunofluorescence test [IFAT] 18/209 dogs), *L*. *infantum* 5% (14/260 dogs; PCR 5/80, IFAT or enzyme linked immunosorbent assay [ELISA] 11/251 dogs), *Babesia* spp. 5% (11/232 dogs; *Babesia* spp. PCR 3/127, *B. canis/vogeli* IFAT or ELISA 8/160, *B. gibsoni* IFAT 2/22), *Dirofilaria* spp. 1% (1/133 dogs; *D. immitis* antigen-ELISA 1/117, microfilariae PCR 0/16, Knott´s test 0/69 dogs). None of the dogs has been tested positive in a combined *Babesia* spp./*Hepatozoon* spp. PCR test (0/15 dogs) and for *H. canis* (0/17 dogs; PCR) or *A. platys* (0/11 dogs; PCR).

There is a substantial risk for dogs travelling to areas endemic for vector-borne pathogens even with limited time of exposure to get infected. The data indicates the importance of owner education and prophylactic measurements against vector-borne infections in dogs travelling to endemic areas.

## Highlights

• There is a risk even for dogs with limited time of exposure to obtain a vector-borne infection.
• Prevention of vector-transferred pathogens in dogs travelling to endemic areas is important.
• The owners should be educated regarding the vectors, diseases, risks and prophylaxis.

## 1 Introduction

Blood-sucking arthropods transmit viral, bacterial and parasitic pathogens that can cause infections and clinical signs in the affected host (ESCCAP, 2019). The spreading of these vector-borne infections depends on the geographic occurrence of the vectors and reservoirs (Shaw et al., 2001). Originally, pathogens such as *Leishmania* (*L*.) spp., *Dirofilaria* (*D.*) spp., *Babesia* (*B.*) *canis/vogeli, B. gibsoni*, *Hepatozoon* (*H.*) *canis*, *Ehrlichia* (*E.*) *canis* and *Anaplasma* (*A.*) *platys* were considered endemic only in the southern and southeastern regions of Europe. Due to the increasing import of dogs from abroad as well as due to the growing tourism and freight traffic, these non-endemic pathogens are brought to Germany. Also, non-endemic vectors that normally only occur in Mediterranean-type climatic regions are imported to more northern countries *e.g.* Germany. Climate changes might allow for these vectors to survive all over the year in central and northern Europe and to establish transmission cycles, for example in the case of *Dirofilaria* (Genchi et al., 2009, Sassnau et al., 2013). Concerning *Leishmania*, *Phlebotomus (P.) mascittii* developed autochthonous sand fly populations in central and western Europe, but the vector competence is up to now questionable (Depaquit et al., 2005, Ready, 2010, Naucke et al., 2011, Kasbari et al., 2012, Obwaller et al., 2016). Endemic vectors can be infected with non-endemic pathogens and may serve as alternate competent vectors by blood-feeding on a naive dog. Autochthonous infections with *B. canis* have been verified in several regions in Germany (Gothe and Wegerdt, 1991, Zahler et al., 2000a, Zahler et al., 2000b, Jensen and Nolte, 2005, Barutzki et al., 2007), amongst others in Berlin-Brandenburg (Heile et al., 2006, Weingart et al., 2017). Individual cases of autochthonous infections with *D. repens* in Germany have been published (Sassnau et al., 2009). Two American pit bull terrier with autochthonous *B. gibsoni* infection and unknown source of infection have been described in southern Germany (Hartelt et al., 2007).

Dogs accompanying their owners on holiday travels to regions, which are endemic for vector-borne pathogens, are at risk of infection and clinical disease. The diagnosis of these vector-borne infections may prove difficult due to the long incubation periods and non-specific clinical signs, and dogs may be infected with multiple pathogens simultaneously (Mekuzas et al., 2009, Cortese et al., 2011). Only a few studies analyzed the test results for vector-borne infections from German dogs travelling to countries being endemic for the transmitting vectors retrospectively (Glaser and Gothe, 1998, Hirsch and Pantchev, 2008, Menn et al., 2010, Hamel et al., 2011, Röhrig et al., 2011, Csokai et al., 2017, Vrhovec et al., 2017) and prospectively (Hamel et al., 2013). The objective of this study was to describe the prevalence of vector-borne infections in dogs from Germany that had travelled to regions in the Mediterranean and southeastern Europe, which are considered as endemic for the mentioned vector-borne pathogens, and were thereafter presented at the Small Animal Clinic at Freie Universität (FU) Berlin.

## 2 Methods

Dogs presented to the Small Animal Clinic at FU Berlin between January 2007 and December 2018 were included into the retrospective study. Inclusion criteria were a stay abroad in an endemic country (13 countries in the Mediterranean area, 4 countries in southeastern Europe) and implementation of at least one test for a vector-borne infection. The dogs were identified *via* keyword search in the clinic’s software program. Medical records and test results were retrospectively analysed for vector-borne infections. Direct and indirect methods of detection were included (Table 1). Direct testing methods detected the pathogen *via* polymerase chain reaction (PCR), antigen-enzyme linked immunosorbent assay (Ag-ELISA) or Knott’s test. Indirect testing methods included the detection of antibodies *via* indirect immunofluorescence test (IFAT) or enzyme linked immunosorbent assay (ELISA) (Table 1). Descriptive statistical analysis was ascertained *via* SPSS for Windows (Version 25.0, SPSS Inc., Armonk, NY, USA). The chi-squared test was used for the comparison of categorical variables. Results were stated in percent and the statistical level of significance was defined as *P* <  0.05.

**Table 1:** Direct and indirect methods of detection for vector-borne infections initiated in travelling dogs.

Infectious agent	Test	LMU Munich	Laboklin
*Ehrlichia canis*	PCR	Applied Biosystems TaqMan^©^ Real Time PCR (Messerer, 2006)	TaqMan^©^ Real Time PCR (in-house test)
	Ab-IFAT	MegaScreen^©^ FLUOEHRLICHIA canis (MegaCor Diagnostik GmbH, Hörbranz, Austria; ≥ 1:40 positive)	MegaFLUO^©^ EHRLICHIA canis (MegaCor Diagnostik GmbH, Hörbranz, Austria; ≥ 1:80 positive)
*Anaplasma platys*	PCR	Applied Biosystems TaqMan^©^ Real Time PCR (Teglas et al., 2005)^a^	TaqMan^©^ Real Time PCR (in-house test)
*Leishmania infantum*	PCR	Applied Biosystems TaqMan^©^ Real Time PCR (Mary et al., 2004)	TaqMan^©^ Real Time PCR (Francino et al., 2006)
	Ab-IFAT	*Leishmania infantum* MON-1 (Mancianti et al., 1995); ≥ 1:64 positive	MegaFLUO^©^ LEISH (MegaCor Diagnostik GmbH, Hörbranz, Austria; › 1:64 positive)
	Ab-ELISA	–	Civtest^©^ Canis Leishmania (Hipra, Amer, Spain; › 1,1 LE positive)
*Babesia* spp.	PCR^b^	PCR (*18S* rRNA) with gel electrophoresis (Casati et al., 2006)^c^	PCR (*18S* rRNA) with gel electrophoresis (Zahler et al., 1998)^d^
*Babesia canis*^e^	Ab-IFAT	MegaScreen^©^ FLUOBABESIA canis (MegaCor GmbH, Hörbranz, Austria; ≥ 1:64 positive)	MegaFLUO^©^ BABESIA canis (MegaCor GmbH, Hörbranz, Austria; ≥ 1:40 positive)
	Ab-ELISA	–	Babesia ELISA Dog (Afosa, Blankenfelde-Mahlow, Germany; 19 TE positive)
*Babesia gibsoni*	Ab-IFAT	MegaScreen^©^ FLUOBABESIA gibsoni-Testkit (MegaCor GmbH, Hörbranz, Austria; ≥ 1:64 positive)	MegaFLUO^©^ BABESIA gibsoni (MegaCor GmbH, Hörbranz, Austria; ≥ 1:32 positive)
*Babesia* spp./ *Hepatozoon* spp.	PCR^b^	In-house protocol^f^	–
*Hepatozoon canis*	PCR	PCR (*18S* rRNA) with gel electrophoresis (Inokuma et al., 2002)^g^	TaqMan^©^ Real Time PCR (in-house test)
*Dirofilaria* spp.	Knott´s test	Modified Knott's test (Rommel et al., 2000)	Modified Knott's test (Rommel et al., 2000)
Microfilariae	PCR	PCR (IST-2) with gel electrophoresis (Rishniw et al., 2006)^c^	TaqMan^©^ Real Time PCR (in-house test)
*Dirofilaria immitis*	Ag-ELISA	Dirochek^©^ Canine Heartworm Antigen Test Kit (Synbiotics Corporation, San Diego, California 92127, US Veterinary License No. 312; Megacor)	FASTest^©^ HW Antigen (MegaCor GmbH, Hörbranz, Austria)

Abbreviations: LMU Munich, Institute for Experimental Parasitology, Ludwig-Maximilians-University Munich, Germany; Laboklin, Laboklin, Bad Kissingen, Germany; PCR, polymerase chain reaction; Ag-ELISA, antigen enzyme-linked immunosorbant assay; Ab-IFAT, immunofluorescence antibody test; Ab-ELISA, antibody enzyme-linked immunosorbant assay.

a In combination with *A. phagocytophilum* PCR due to sequence homology.

b Differentiation between different species possible by request of veterinarian.

c Species differentiation after sequencing of the PCR product and comparison with the database GenBank (NCBI Blast Search).

d Sequencing of the PCR-product by request of the veterinarian.

e Serological cross-reactions between *B. canis* und *B. vogeli* possible.

f Only available in the year 2008.

g *18S* rRNA, 2012–2015 (2007–2012 no data available).

## 3 Results

### 3.1 Signalment/History

Three hundred and three dogs with a travel history involving 14 endemic countries for the infections L. *infantum*, *H. canis*, *E. canis, A. platys, Babesia* spp. and *Dirofilaria* spp. were included into the study (Table 2). Most dogs had accompanied their owners to Italy (90/303, 30%), France (53/303, 17%) or Spain (49/303, 16%). A total of 57/303 dogs (19%) had visited more than one country endemic for the mentioned vector-borne pathogens. Fourty-nine dogs had travelled to two countries, eight dogs to three countries.

**Table 2:** Number of vector-borne infections in dogs travelling to countries endemic for vector-borne pathogens (number of monoinfections/number of multiple infections).

Holiday country	No. of dogs tested positive/total (%)	*E. can*	*L. inf*	*B.* spp.^a^	*B. can*^b^	*B. gib*	*D.* spp.	Coinfections positive/total (%)
Italy	13/90 (14)	4/1	3/1	1/-	3/-	1^c^/-	-/-	*E. can + L. inf*
France	5/53 (9)	1/1	2/1	-/-	-/1	-/-	0/1^d^	*B.* spp. *+ L. inf; E. can + D.* spp.
Spain	6/49 (12)	2/-	4/-	-/-	*-/-*	-/-	-/-	–
Croatia	3/15 (20)	2/-	-/-	-/-	1/-	-/-	-/-	–
Hungary	0/10 (0)	-/-	-/-	-/-	-/-	-/-	-/-	–
Greece	2/8 (25)	1/-	1/-	-/-	-/-	-/-	-/-	–
Turkey	3/6 (50)	2/-	-/-	-/-	1/-	-/-	-/-	–
Portugal	1/6 (17)	-/1	-/-	-/-	-/1	-/-	-/-	*B.* spp. *+ E. can*
Romania	2/4 (50)	-/-	1/-	1/-	-/-	-/-	-/-	–
Serbia	1/3 (33)	1/-	-/-	-/-	-/-	-/-	-/-	–
Bulgaria	0/2 (0)	-/-	-/-	-/-	-/-			–
2 countries	4/49 (8)	2/-	1/-	-/-	-/-	1^e^/-	-/-	–
3 countries	0/8 (0)	-/-	-/-	-/-	-/-	-/-	-/-	–
Total	40/303 (13)	15/3	12/2	2/-	5/2	2/-	0/1	4/303 (1)

Abbreviations: E. can, *Ehrlichia canis*; L. inf, Leishmania infantum; B. spp., Babesia spp.; B. can, Babesia canis; B. gib, Babesia gibsoni; D. spp., Dirofilaria spp.

a Not differentiated *Babesia* spp. PCR (polymerase chain reaction).

b Serological cross-reactions between *B. canis* and *B. vogeli* possible.

c Positive *B. gibsoni* IFAT without species differentiation *via* PCR.

d Positive *D. immitis* Ag-test.

e Detection of *B. gibsoni* after species differentiation *via* PCR.

One hundred and fourty-five of 303 dogs (48%) were males, 158 (52%) were females. One hundred eighty-two of 303 dogs (60%) were crossbreeds and 121/303 (40%) were purebreds. The age was known in 302/303 dogs with a median of 8 years (0.5–14.9 years). Two hundred and eighty-eight of 303 dogs (95%) were presented with clinical signs and 15/303 dogs (5%) for a health check. The time between the last stay abroad and presentation in the clinic is listed in Table 3. Most of the dogs with clinical signs had been presented 1–5 years after staying abroad (95/288, 33%), followed by 6 months to 1 year (43/288, 15%), 2–6 months (26/288, 9%), 0–2 months (21/288, 7%), >7 years (15/288, 5%) and 5–7 years (6/288, 2%). In 82/288 (28%) dogs with clinical signs no time period could be determined. Eight of 172 dogs (5%) that were presented between >2 months and <5 years after their stay abroad and 7/89 dogs (8%) in which it was not able to determine the exact period of time, were presented for a health check-up and did not show any clinical signs, representing 6% (15/261 dogs) in total.

**Table 3:** Number of dogs tested positive for vector-borne infections sorted by time interval between travelling to countries endemic for vector-borne pathogens and presentation in the clinic.

Period	Positive/total (%)	Monoinfections			Coinfections
		*E. canis*	*L. infantum*	*B.* spp.	
No data	8/89 (9)	2	3	1	2 (*E. canis* + *L. infantum, E. canis* + *B.* spp.)
0-2 months	6/21 (29)	2	2	2	–
2-6 months	3/28 (11)	–	1	1	1 (*B.* spp + *L. infantum*)
6-12 months	6/45 (13)	3	1	2	–
1-5 years	13/99 (13)	6	4	2	1 (*E. canis* + *D.* spp.)
5-7 years	2/6 (33)	1	–	1	–
› 7 years	2/15 (13)	1	1	–	–
**Total**	**40/303 (13)**	**15**	**12**	**9**	**4**

### 3.2 Laboratory diagnostics

A total of 1174 tests for vector-borne infections were analysed between January 2007 and December 2018. Twelve of 525 (2%) direct detection methods and 39/649 (6%) indirect detection methods were positive (Table 4). *Ehrlichia canis* was detected in 18/231 dogs (8%) and L. *infantum* in 14/260 dogs (5%). Eleven out of 232 dogs (5%) were positive for *Babesia* (2 dogs with a positive PCR result without further differentiation, 1 dog with a differentiated PCR detecting *B. gibsoni* and additionally with a positive *B. gibsoni* IFAT and *B. canis/vogeli* ELISA, 2 dogs with a positive *B. canis/vogeli* IFAT result and negative PCR, 2 dogs with positive *B. canis/vogeli* IFAT results, 3 dogs with positive *B. canis/vogeli* ELISA in combination with two negative and one invalid PCR result, 1 dog with positive *B. gibsoni* IFAT and negative *B. canis/vogeli* IFAT). One dog was positive for *D. immitis* (Ag-ELISA) and was coinfected with *E. canis*. None of the dogs was tested positive for *H. canis*, *A. platys*, or a combined *Babesia* spp./*Hepatozoon* spp. PCR.

**Table 4:** Number of positive tests for vector-borne infections in 303 dogs travelling to countries endemic for vector-borne pathogens.

Infectious agent/test	No. of dogs tested positive/total (%)	Direct tests (positive/total)	Indirect tests (positive/total)
*Ehrlichia canis*	18/231 (8)	3/73^a^	18/209^b^
*Anaplasma platys*	0/11 (0)	0/11^a^	-/-
*Leishmania infantum*	14/260 (5)	5/80^a^	9/215^b^, 2/38^c^
*Babesia* spp.	3/127 (2)	3/127^a^, ^d^	-/-
*Babesia canis*^e^	8/160 (5)	–	4/141^b^, 4/24^c^
*Babesia gibsoni*	2/22 (9)	–	2/22^b^
*Babesia* spp.*/* *Hepatozoon* spp.	0/15 (0)	0/15^a^	-/-
*Hepatozoon canis*	0/17 (0)	0/17^a^	-/-
*Dirofilaria immitis*	1/117 (1)	1/117^f^	-/-
Microfilariae	0/16 (0)	0/16^a^	-/-
Modified Knott´s test	0/69 (0)	0/69	-/-
**Total**	**40/303 (13%)**	**12/525 (2%)**	**39/649 (6%)**

a Polymerase chain reaction.

b Immunofluorescence antibody test.

c Antibody enzyme-linked immunosorbant assay.

d 1/3 positive PCR test differentiated as *B. gibsoni*.

e Serological cross-reactions between *B. canis* und *B. vogeli* possible.

f Antigen enzyme-linked immunosorbant assay.

Vector-borne infections were most frequently found in dogs with a stay in one of the following countries taken: Croatia (3/15 dogs, 20%), Italy (13/90 dogs, 14%) and Spain (6/49 dogs, 12%). This comparison only considers the countries that were visited by 10 or more dogs. Four out of 49 dogs (8%) that travelled to two endemic countries were tested positively for a vector-borne infection. Two dogs with *E. canis* had visited Italy/Croatia and Italy/Greece, respectively. One dog with *L*. *infantum* and one dog with *B. gibsoni* had been in Italy/France. None of the eight dogs that had travelled to three countries were positively tested for a vector-borne infection. Coinfections with two pathogens were detected in 4/303 dogs (1%) (Table 2).

**Fig. 1:**
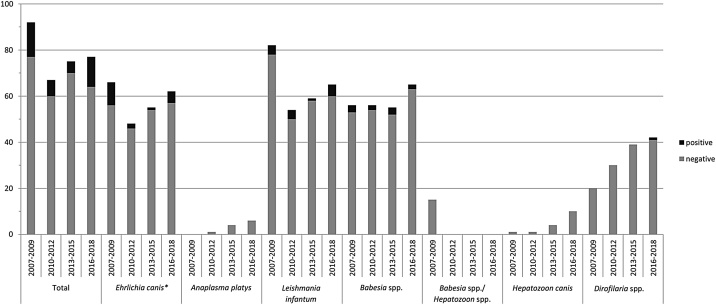
Number of travelling dogs tested for vector-borne infections between 2007 and 2018. *P = 0.035 for number of dogs tested positive from 2007 to 2018 (the statistical level of significance was defined as *P* <  0.05).

The number of dogs tested positive during the periods 2007–2009, 2010–2012, 2013–2015 and 2016–2018 differed with statistical significance for *E. canis* (*χ^2^* = 8.591; *df* = 3; *P* =  0.035). The difference was not significant for L. *infantum* (*χ^2^* = 2.731; *df* = 3; *P* =  0.435) and *Babesia* spp. (*χ^2^* = 0.281; *df* = 3; *P* =  0.964). The number of tests initiated for *A. platys*, *Babesia* and *Dirofilaria* increased when comparing the periods 2007–2009, 2010–2012, 2013–2015 and 2016–2018 (Figure 1).

## 4 Discussion

Out of 303 dogs that accompanied their owners to endemic countries and that were tested for vector-borne infections, 13% were positive for at least one pathogen. Infections with *E. canis* (8%), L. *infantum* (5%), *Babesia* spp. (5%) and *D. immitis* (1%) could be detected (Table 2). There was no significant change concerning the number of *Babesia* and *Leishmania* infections during the study period, but a mild decrease of *E. canis* infections from 2007 to 2015. Possible explanations could be the improved education of patient owners *via* media and/or veterinarians resulting in increased utilization of prophylactic measures for travelling dogs, and further development of preventive medications. The number of dogs that were tested for *A. platys*, *H. canis* and *Dirofilaria* spp. increased during the study period (Figure 1). The higher number of tests performed possibly is due to the fact that there existed a rising awareness of these infectious pathogens resulting in an increase of testing.

For correct diagnosis and adequate treatment, one must differentiate between the contact with a pathogen, infection with a pathogen and clinical disease. The differentiation of pathogen contact/infection and clinical disease is based on clinical presentation as well as laboratory results and the exclusion of differential diagnosis. The sensitivity of direct and indirect detection methods is variable for each of the pathogens and also depends on the sample material. PCR or Ag-ELISA are direct methods to detect antigens respectively deoxyribonucleic acid (DNA) of the infectious agents. Dogs which are tested positive can be classified as infected. PCR testing is recommended in early still seronegative stages of infection and in puppies, due to the existence of maternal antibodies (Pantchev et al., 2017). However, a detectable amount of antigen or DNA must be present in the tested sample material (Hernandez et al., 2015), meaning that a negative test result does not necessarily exclude an infection. Indirect methods of detection reveal antibodies towards a pathogen. It is not possible to differentiate between pathogen contact and infection *via* a single test. Acute infection is likely if the antibody titer increases or decreases four-fold within the follow-up examination. Usually, IFAT and ELISA have a high sensitivity and specificity, but the limitations of serological tests lie within the cross-reactions with other pathogens, false-negative results in young or immunosuppressed dogs as well as the premature implementation of tests post infection before the beginning of seroconversion (Solano-Gallego et al., 2014). Seroconversion in *Leishmania* can occur years after the initial contact (ESCCAP, 2019) and *Dirofilaria* have a long prepatency (Pantchev et al., 2017), meaning that it is recommended to repeat negative test results for these pathogens six months after returning to Germany from travelling to endemic countries (Pantchev et al., 2017). For the detection of different cyclic stages of *Dirofilaria*, enrichment methods such as the Knott’s test or a microfilariae PCR can be combined with a *Dirofilaria* antigen test. Due to the late seroconversion in *Leishmania* and the long prepatency for *Dirofilaria*, it is possible that the infection rates for these pathogens (*Dirofilaria*: 1%; *Leishmania* 5%) are actually higher than determined in this study. In addition to laboratory diagnostics, information on prophylactic measures, clinical signs and the region the dog had travelled to are of great importance. For example, there are great differences in the prevalence of vector-borne infections in France between the Mediterranean regions in the south and the northern parts of the country, *e.g.* for *L*. *infantum* (Chamaille et al., 2010), *D. immitis* (Pantchev et al., 2009) and *Babesia* spp. (Halos et al., 2013).

The spreading of vector-borne infections is not only influenced by biotic and abiotic factors, but also by the prevalence of the transmitting vector. *Rhipicephalus* (*R.*) *sanguineus* is considered to be the vector for *B. vogeli*, *B. gibsoni*, *E. canis*, *A. platys* and *H. canis* and can therefore trigger more than one infection in the regarding host (ESCCAP, 2019). *Rhipicephalus sanguineus* can only temporarily survive in northern countries, as for example Germany, when specific temperature ranges are present or as a population in all year heated premises (Pantchev et al., 2015). Therefore, the 18 dogs infected with *E. canis* in this study were most likely infected whilst staying in an endemic region. *Leishmania infantum* is transmitted *via Phlebotomus* spp. In the past this vector was mostly found in the Mediterranean area but has also been detected in Germany. The vector competence has been confirmed for *P. perniciosus*, which has been detected in southern Germany (Naucke and Schmitt, 2004). Another species, *P. mascittii*, was found in Hesse (Melaun et al., 2014), but its vector competence is questionable (Obwaller et al., 2016). Currently, no *Phlebotomus* spp. with proven vector competence have been detected in Berlin-Brandenburg. Literature not only describes transmission *via* vectors, but also individual cases of transplacental infections (Gibson-Corley et al., 2008, Petersen, 2009, Boggiatto et al., 2011), infections *via* mating (Benites et al., 2011, Turchetti et al., 2014) or infections transmitted *via* bite wounds (Naucke et al., 2016), but these are most likely not epidemiologically relevant in the case of travelling dogs in the present study. Vaccinations cause an immune response with development of antibodies and can thereby cause a positive result *via* indirect detection methods. The European Commission approved the adjuvanted vaccine CaniLeish^©^ against L. *infantum* for dogs on the 14th of March 2011. Theoretically this could relate to three dogs in this study that were tested positive *via* IFAT in the years 2013, 2016 and 2017, and to two dogs tested positive *via* ELISA in the years 2017 and 2018. In none of the dogs a vaccination was mentioned in the medical records.

Out of 232 dogs tested for *Babesia* in the present study, eleven (5%) had a positive result. Indirect methods of detection do not allow for a further differentiation between *Babesia* spp. because of serological cross-reactions, which is why an additional *Babesia* PCR is recommended for species differentiation. *Babesia* spp. being endemic in Europe include *B. canis*, *B. vogeli* and *B. gibsoni*. Autochthonous infections with *B. canis* have been described in several regions throughout Germany (Gothe and Wegerdt, 1991, Zahler et al., 2000b, Jensen and Nolte, 2005, Jensen et al., 2007), including Berlin-Brandenburg (Heile et al., 2006, Weingart et al., 2017). In these regions *Dermacentor reticulatus* is considered to be the vector. Generally, *B. canis* occurs more frequently in central and western Europe (Halos et al., 2013), but has also been detected in the Mediterranean (Solano-Gallego et al., 2016). As part of a questionnaire-based survey, 225 dogs in Germany were identified that had been autochthonously infected with *B. canis*, including three dogs from Berlin-Brandenburg (Barutzki et al., 2007). *Babesia vogeli* is transmitted *via R. sanguineus* and is mainly endemic in the Mediterranean due to the prevalence of the vector. Thus in the Mediterranean an infection with *B. vogeli* is more likely than one with *B. canis* (Solano-Gallego et al., 2016). For 9/11 dogs tested *Babesia* positive, a pathogen contact/infection with *B. canis/vogeli* seems most likely. Two of these nine dogs were positive in a non differentiated *Babesia* PCR. One of these two dogs had travelled to Italy two years and to Poland one week prior to presentation, the second dog had travelled to Romania one week prior to presentation. Both dogs had tick contact whilst staying abroad and had acute clinical and clinicopathological signs. An infection with *B. canis* in Poland and in Romania seems to be most likely because of the acute onset of disease. One dog that was positively tested *via* IFAT had been to Croatia a couple of days before presentation and was presented with tick infestation as well as acute clinical signs. A *Babesia* infection in Croatia seems most likely. In three dogs (one with a positive *B. canis/vogeli* ELISA, two with a positive *B. canis/vogeli* IFAT) no laboratory abnormalities and in one dog tested positive *via B. canis/vogeli* IFAT a mild thrombocytopenia were detected and pathogen contact might have occurred in Germany with *B. canis* and/or whilst staying abroad with *B. canis/vogeli*. Two dogs with a positive *B. canis/vogeli* ELISA result lived together in one household and had been to Italy one year prior to presentation. The owners reported infestation with ticks in Germany. In both dogs an infection within Germany is possible. Nevertheless, pathogen contact whilst staying abroad cannot be excluded.

Two of the eleven dogs tested *Babesia* positive seem to be infected with *B. gibsoni*. Infections with *B. gibsoni* are considered rare in Europe (ESCCAP, 2019). For example, infections have been reported in Italian dogs (Trotta et al., 2009). In Germany autochthonous infections were detected in two American pit bull terriers from southern parts of the country with unknown source of infection (Hartelt et al., 2007). *Rhipicephalus sanguineus* has been discussed as a possible vector in Europe, but the vector competence has not been confirmed (Birkenheuer, 2012). Next to vector-contact, possible routes of infection are *via* dog biting (Jefferies et al., 2007), *via* blood transfusion (Stegeman et al., 2003) or transplacental (Fukumoto et al., 2005). One of the dogs in the present study that had travelled to Italy and France had a positive *B. gibsoni* PCR, a positive *B. gibsoni* IFAT and a positive *B. canis* ELISA. Another dog which had stayed in Italy had a positive *B. gibsoni* IFAT and a negative *B. canis* ELISA. Pathogen contact whilst staying abroad seemed most likely for these two dogs.

Coinfections with two pathogens were detected in 4/303 dogs (1%). All affected dogs had been to countries with direct access to the Mediterranean Sea. *Ehrlichia canis* and/or *Babesia* spp. were involved in all four dogs (*E. canis* + L. *infantum*, *B. canis/vogeli* + L. *infantum*, *B. canis/vogeli* + *E. canis*, *Dirofilaria* spp. + *E. canis*). *Leishmania infantum* and *E. canis* can induce an immunosuppression, which can result in infection with further pathogens (Nyindo et al., 1980, Adachi et al., 1993). Because three of the four dogs were diagnosed only *via* positive serological methods of detection, there are two possibilities: they were either infected with two pathogens or the multiple positive results were due to serological cross-reactions.

Although a few retrospective studies analysing vector-borne infections in dogs from Germany with stays abroad have been published (Hirsch and Pantchev, 2008, Menn et al., 2010, Hamel et al., 2011, Csokai et al., 2017, Vrhovec et al., 2017), a comparison between those studies and the present study proves difficult for example because of a varying spectrum of included pathogens and holiday countries. In the study of Hamel et al. (2011), the number of positive direct detection methods (3.5%) was lower compared with the number of positive indirect methods of detection (7.5%). This was the same in the present study and can be explained by the fact that antibodies can persist for a long time. In both studies no infections with *H. canis* were detected. Hamel et al. (2011) determined lower prevalences for *E. canis* and L. *infantum*, but they had also included non-endemic countries for these pathogens *e.g.* Belgium, Netherlands, Russia or Scandinavian countries. The number of infections with *Babesia* spp. (1%) and the number of co-infections (1%) was comparable with the present study. In the study of Menn et al. (2010), the percentage of dogs accompanying their owners on travels was 1.8% and therefore very low. They also included *A. phagocytophilum* in their evaluation with a high seroprevalence of 22.4%. However, an infection with this pathogen seems more likely in Germany than in countries of the Mediterranean area (Kohn et al., 2011). Another retrospective study analysed 5483 dogs that had been imported or had travelled to countries abroad, however the study did not evaluate the differences between the two groups (Hirsch and Pantchev, 2008).

In a prospective study from 2012 106 dogs from Germany were analysed in order to determine the risk of infection for *Babesia* spp., *Leishmania* spp. and *E. canis* after travelling to a region endemic for these vector-borne pathogens (Hamel et al., 2013). Seven out of 106 dogs (6.6%) had been tested positive for a vector-borne infection before travelling, but all 7 dogs had been to an endemic region before participating in the trial. Following this, the dogs travelled to endemic countries in south and southeastern Europe for an average of 17 days and were tested for vector-borne infections at three different time points after their return (2–4 weeks, 6–8 weeks, 6 months). No infection was determined after their return, however 51% of the study population had undergone prophylactic treatment before travelling. The authors concluded that the individual risk for a dog is low when visiting an endemic country for a limited time. Another study in the Netherlands tested 434 dogs serologically for L. *infantum*, which travelled to southern Europa up to three years ago. None of these dogs was tested positive and a minimal risk of infection has been concluded (Teske et al., 2002).

Imported dogs in Germany had a considerably higher prevalence for vector-borne infections (Menn et al., 2010; Röhrig et al., 2011; Schäfer et al., 2019) compared with the German dogs with travel history. Imported dogs were mostly strays that had received no or little veterinary support in their home countries. Therefore, prophylactic measures against vector-borne infections were not implemented. Furthermore, imported dogs have usually stayed in regions, which are endemic for vector-borne pathogens, for a long period of time, thereby increasing the likeliness of vector contact and – consequently - the risk of infection.

In our retrospective study, information regarding prophylactic measurements before and during their stays abroad could not be included because of missing information in the medical records. Prophylactic measures might have influenced the prevalence rates of the different vector-borne infectious pathogens. Moreover it was not exactly known in which month the dogs had been abroad and seasonal variations could have influenced primarily the incidence and secondly also the prevalence rates of the different vector-borne infectious pathogens. In the present study the different countries were considered in the analyses, but not the various regions within the endemic countries. Another limitation of the study lies in the retrospective study design, but also in the fact that not all tests were performed on every dog. Furthermore, the duration of the stay abroad was not determined. The precision of the detection methods was enhanced and improved during the study interval between 2007 and 2018. Regardless of the limitations of this study, 13% of 303 dogs have been tested positive for at least one vector-borne infection. The data emphasizes the necessity to prophylactically protect all dogs against vector-borne infections, irrespective of origin and current residence, especially considering the increasing tourism within Europe and the spreading of potentially competent vectors. Because some pathogens like *L*. *infantum*, *D. immitis* and *D. repens* have zoonotic potential, prophylactic arrangements are not only of importance for animal health, but also for human medicine and the public health in Europe (ESCCAP, 2019).

## 5 Conclusions

Thirteen percent of dogs that had travelled to endemic countries were tested positive for at least one vector-borne pathogen. Coinfections with two pathogens were found in 1% of dogs. The analysed data reveal that risk of infection also exists when the stay occurs for a limited time. This study highlights the importance of owner education and prophylaxis against vector-borne infections in dogs travelling to endemic areas but also living in non-endemic regions.

## Ethics approval and consent to participate

Not applicable.

## Consent for publication

Not applicable.

## Availability of data and materials

All data generated during this study are included in this published article. Parts of this study were presented as a poster / oral presentation at the 28th Annual Meeting of the German Society for Parasitology in Berlin, Germany (21–24 March 2018), the 64th DVG-Congress in Berlin (04-07 October 2018) and the DVG-Congress for Internal Medicine and Laboratory Diagnostics in Hannover, Germany (02–03 February 2018).

## Funding

This research did not receive any specific grant from funding agencies in the public, commercial, or not-for-profit sectors.

## Authors’ contributions

IS collected and evaluated the data and wrote the manuscript. BK initiated and supervised the study and edited the manuscript. MV and RM supported the statistical analyses and edited the manuscript. EM and PB were responsible for laboratory analyses and edited the manuscript. All authors read and approved the final manuscript.

## Declaration of Competing Interest

None.
